# Differences in nasal immunoglobulin A responses to influenza vaccine strains after live attenuated influenza vaccine (LAIV) immunization in children

**DOI:** 10.1111/cei.13395

**Published:** 2019-11-15

**Authors:** P. J. Turner, A. F. Abdulla, M. E. Cole, R. R. Javan, V. Gould, M. E. O'Driscoll, J. Southern, M. Zambon, E. Miller, N. J. Andrews, K. Höschler, J. S. Tregoning

**Affiliations:** ^1^ National Heart and Lung Institute Imperial College London London UK; ^2^ Public Health England (Colindale) London UK; ^3^ Department of Infectious Disease St Mary's Campus Imperial College London London UK; ^4^ Infectious Diseases Epidemiology St Mary's Campus Imperial College London London UK

**Keywords:** children, influenza, nasal, public health, vaccine

## Abstract

Different vaccine strains included in the live attenuated influenza vaccine (LAIV) have variable efficacy. The reasons for this are not clear and may include differences in immunogenicity. We report a Phase IV open‐label study on the immunogenicity of a single dose of quadrivalent LAIV (Fluenz™ Tetra) in children during the 2015/16 season, to investigate the antibody responses to different strains. Eligible children were enrolled to receive LAIV; nasal samples were collected before and approximately 4 weeks after immunization. There was a significant increase in nasal immunoglobulin (Ig)A to the H3N2, B/Victoria lineage (B/Brisbane) and B/Yamagata lineage (B/Phuket) components, but not to the H1N1 component. The fold change in nasal IgA response was inversely proportional to the baseline nasal IgA titre for H1N1, H3N2 and B/Brisbane. We investigated possible associations that may explain baseline nasal IgA, including age and prior vaccination status, but found different patterns for different antigens, suggesting that the response is multi‐factorial. Overall, we observed differences in immune responses to different viral strains included in the vaccine; the reasons for this require further investigation.

## Graphical abstract

Different vaccine strains included in the Live Attenuated Influenza Vaccine (LAIV) have variable efficacy. We performed an immunogenicity study of quadrivalent LAIV in children during the 2015/16 season. There was a significant increase in nasal IgA to the H3N2, B/Victoria lineage (B/Brisbane), and B/Yamagata lineage (B/Phuket) components, but not to the H1N1 component.

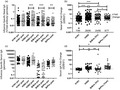

## Introduction

Influenza infection is a significant cause of morbidity and mortality worldwide; the World Health Organization (WHO) estimates that there are 3–5 million severe cases every year, causing 290 000–650 000 deaths globally 1. Vaccination remains the main preventative strategy, but efficacy is highly dependent upon the correct matching of the vaccine strains to the circulating viruses. The vaccine strains are selected twice a year, in February for the northern hemisphere and September for the southern hemisphere. Strain selection is based on surveillance data to identify which strains are circulating and their antigenic differences from historic strains, as determined by changes in gene sequence and haemagglutination inhibition 2. The most common vaccine used is inactivated influenza vaccine (IIV) administered by injection, but this provides variable levels of protective efficacy, ranging from 10% in 2004–05 3 to 60% in 2010–11 4. An alternative is the live attenuated influenza vaccine (LAIV), which includes cold‐adapted attenuated influenza strains and is administered as a nasal spray. This replicates locally in the air‐cooled upper respiratory tract resulting in a mild, subclinical self‐limiting infection that does not extend to the warmer lower respiratory tract. The route of administration for LAIV is particularly well‐suited to the vaccination of children.

The United Kingdom has implemented the phased introduction of one of the first publicly funded universal influenza vaccination programmes for children using LAIV 5, at a cost of approximately £100 million per year. The programme is based on cost‐effectiveness analyses 6, with a particular emphasis on targeting younger children, who act as a reservoir for potential spread to elderly people. Under the national programme children are immunized annually, receiving a single dose of LAIV even if receiving the vaccine for the first time. However, questions concerning the efficacy of LAIV have arisen. The reported vaccine effectiveness for LAIV in the United States has declined significantly since the emergence of the 2009 swine‐origin pandemic H1N1 (pH1N1). Before 2009, LAIV showed 85% efficacy against seasonal H1N1 strains in children, falling dramatically to 17% in the 2013–14 7 and 3% in the 2015–16 seasons 8; this led the Advisory Committee on Immunization Practices (ACIP) to recommend suspension of LAIV in the United States for the 2016–17 and 2017–18 9 seasons. Both the United Kingdom and Finland continued to use LAIV, as local data demonstrated ongoing vaccine efficacy. The ACIP subsequently approved LAIV for the 2018–19 season, following a further change in the H1N1 component by the manufacturer to A/Michigan/45/2015 10.

Within the United Kingdom, differences have also been observed in vaccine efficacy between A and B strains after LAIV immunization 11. One reason for this may be differences in immunogenicity of the different strains included in LAIV 12. Vaccine immunogenicity may be affected by viral factors including the fitness of the vaccine virus strain and host factors including prior influenza virus exposure, either from vaccination or infection. We therefore undertook a Phase IV open‐label study on the immunogenicity of a single dose of a quadrivalent (Fluenz™ Tetra) in children during the 2015–16 season to investigate the local antibody response to different strains in the quadrivalent LAIV formulation and how this might be affected by baseline (pre‐vaccine) antibody titres. We measured strain‐specific nasal immunoglobulin (Ig)A, which has previously been proposed as a better correlate of protection against influenza infection for LAIV 13.

## Methods

### Study plan

We conducted a Phase IV open‐label study of LAIV in children aged 2–17 years with enrolment occurring during the United Kingdom influenza season (October 2015–January 2016) at a single UK centre (Clinicaltrials.gov registration NCT02549365). The study was approved by the National Health Service (NHS) Health Research Authority, and informed consent/patient assent was obtained. Study participants were recruited at St Mary's Hospital, London. Quadrivalent LAIV (Fluenz Tetra, produced for the 2015–16 influenza season) was administered into the nasal airway according to the approved summary of product characteristics. This vaccine had four strains: two A strains: H1N1 (A/California/7/2009 pdm‐like), H3N2 (A/Switzerland/9715293/2013); and two B strains: Yamagata‐like (B/Phuket/3073/2013) and Victoria‐like (B/Brisbane/60/2008). The H1N1 and B/Bris strains had been used in previous seasons, but the H3N2 and B/Phu strains were new to the 2015–16 northern hemisphere vaccine recommendation. Nasal samples were collected before immunization in the clinic, with participants or their parents requested to take a follow‐up nasal sample approximately 4 weeks (allowable limits = 3–6 weeks) after immunization by a flocked swab (Copan Inc., Murietta, CA, USA) and to return this by post. Families were sent an e‐mail reminder at this time. The brush was vortexed with 300 μl extraction buffer prior to centrifugation in a 0·22 μm Spin‐X column to remove debris; this process was repeated. Where additional consent was obtained, subjects returned after 4 weeks for a further blood sample. Samples were stored at –80°C until assay.

### Preliminary study on stability of posted samples​

Adult volunteers were swabbed with a nasal flocked swab inserted into the nasal cavity. Samples were processed as above and stored at –80°C until required. A second swab was taken and sent through the mail system to replicate a sample being posted from study participants, and processed upon receipt in the same way.

### Haemagglutination inhibition (HAI) assay

Serological analysis was performed at the National Infection Service of Public Health England (London, UK). Antigens for all assays were egg‐grown; influenza B antigens were Tween80/diethyl‐ether extracted prior to their use in haemagglutination inhibition (HAI) assays by adding Tween^®^ 80 to final concentrations of 0·125%, incubation for 20 min at room temperature and addition of a half‐volume of diethyl ether. The mixtures were stirred for a minimum of 45 min inside a fume cupboard and then left standing until the phases had fully separated and the aqueous phase could be extracted for use as antigen. HAI assays were performed using previously described methods 14. Briefly, sera treated to remove non‐specific inhibitors were twofold serially diluted starting at a 1 : 10 dilution, then mixed with an equal volume (25 μl) of phosphate‐buffered saline (PBS) containing four haemagglutinating (HA) units of each of the vaccine strains. Turkey red blood cells (RBC) were used for the influenza A/H1N1pdm09 and influenza B components, and guinea pig RBC for H3N2. HAI titres were expressed as the reciprocal of the last serum dilution that gave complete inhibition of agglutination.

### IgA enzyme‐linked immunosorbent assay (ELISA)

Influenza‐specific antibodies were measured using a standardized ELISA 15. IgA responses were measured in nasal swabs and sera. To detect antigen‐specific responses, Nunc MaxiSorp 96‐well plates (Thermo Fisher Scientific, Loughborough, UK) were coated with 1 μg/ml recombinant haemagglutinin from H1N1 (A/California/09), H3N2 (A/Switzerland/9715293/2013), B/Phuket/3073/2013 and B/Brisbane/60/2008 antigens (Sino Biologicals, Beijing, China) and incubated overnight at 4°C. Plates were blocked with 1% bovine serum albumin (BSA) in PBS, which was also used for washing steps. Bound IgA was detected using a biotinylated anti‐IgA (AbD Serotec, Oxford, UK) followed by poly‐horseradish peroxidase (HRP) 40 (Fitzgerald Biotech, Oxford, UK). To quantify the concentration of antigen‐specific antibody, control wells were coated with a combination of anti‐human lambda and kappa light chain‐specific antibodies (AbD Serotec) and a dilution series of control non‐specific IgA (Sigma, Poole, UK) was used as a standard in these wells. 3,3*'*,5,5*'*‐Tetramethylbenzidine (TMB) with H_2_SO_4_ as stop solution was used to detect the response and optical densities read at 450 nm. Nasal IgA units are given as a percentage of total IgA with a minimum set at 0·01% (all values < 0·01% are set to 0·01%) and a requirement that total IgA is at least 0·3 μg/ml.

### Statistics

Geometric mean antibody concentrations and fold rises were calculated with 95% confidence intervals and proportions with fourfold rises described. Comparison of antibody concentrations pre‐ to post‐vaccination as well as between strains was performed using the signed‐rank test for paired data. Regression on logged data was used to compare baseline nasal IgA (and fold change in nasal IgA) by prior vaccination status. Regression was also used on logged baseline nasal IgA to assess the relationship with age and with logged fold‐change in nasal IgA or serum HAI.

Statistical comparison of HAI and IgA titres before and after immunization and comparison of HAI and fold change in IgA were planned as part of the original study; *post‐hoc* analysis was performed to investigate correlations between age, vaccination status and baseline nasal IgA. For analyses comparing strains, correction was made for six multiple comparisons and for analyses comparing pre‐ and post‐levels for four multiple comparisons. Analysis was performed in Stata version 15 and GraphPad Prism version 8.

## Results

### Posting swabs does not impact influenza specific nasal IgA recovery

We undertook a pilot test to confirm that posting of samples (resulting in a 3‐day delay in processing) had no effect on the recovery of influenza‐specific nasal IgA in six adult volunteers. There were no significant differences in antibody levels measured in the fresh or posted samples to either the H1N1 (paired *t*‐test; *P* = 0·47, Supporting information Fig. S1a) or H3N2 (paired *t*‐test; *P* = 0·42, Supporting information, Fig. S1b) antigens.

### Demographics of cohort for childhood immunization study

Characteristics of the children vaccinated in the study are shown in Table 1. Of the 164 children, recruited, 103 provided paired pre‐ and post‐vaccination samples, and are reported in this paper; in 61 cases, no second nasal swab was received. There were no differences in baseline characteristics between these 103 children and the overall cohort (Table 1). Fifty children (48·5%) had been previously immunized with an influenza vaccine. Paired blood samples pre‐ and post‐vaccine were provided in a subgroup of 39 children. No serious adverse effects were reported.

**Table 1:** Characteristics of the study population who gave nasal samples

Characteristic	Complete cohort *n* = 164	Children included in this analysis *n* + 103	Previously vaccinated *n* = 50	Previously unvaccinated *n* = 53
Female	65 (40%)	37 (36%)	15 (30%)	22 (41·5%)
Number of siblings (median, range)	1 (0–6)	1 (0–6)	1 (0–6)	1 (0–4)
Age, years (median, range)	7 (2–17)	8 (2–16)	9·5 (2–16)*	7·5 (2–15)
2–5		36	16	20
6–11		37	15	22
12–17		30	19	11
Asthma or wheeze	81 (49%)	51 (50%)	32 (64%)**	19 (38%)
Any atopy	116 (71%)	68 (66%)	36 (72%)	32 (60%)
Date of vaccination in study (median month)	November	November	November	November
Previous vaccination (all types)	78 (48%)	50 (49%)	50	–
Previous vaccination (LAIV)	41	24	24	–
Previous vaccination (IIV)	36	20	20	–
Previous vaccination (unknown format)	7	5	5	–
Previous vaccination (Pandemrix only)	1	1	1	–
Paired blood samples collected	42	39	23	16

Age, years, *P < 0·05 by *t*‐test; asthma, **P < 0·01 by Fisher's exact test.

LAIV = live attenuated influenza vaccine.

### LAIV immunization induces local and systemic IgA response to some but not all strains in the vaccine

**Figure 1:**
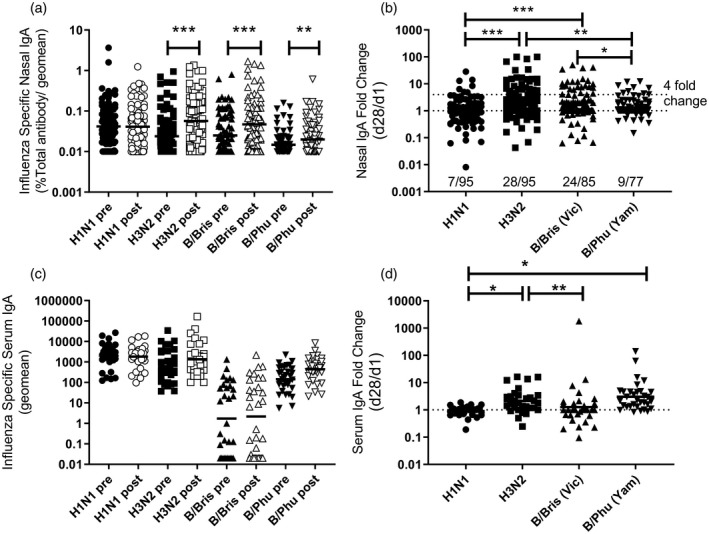
Live attenuated influenza vaccine (LAIV) immunization induces a nasal immunoglobulin (Ig)A response to some, but not all, strains in the vaccine. Children were immunized with LAIV, serum samples were collected from 39 children and nasal samples were collected from 103 children before (pre) and after (post) immunization. IgA titre (a,c) and fold change (b,d) were measured in nasal (a,b) and serum (c,d) samples. *P < 0·05; **P < 0·01; ***P < 0·001. Points represent individuals, with lines representing geomean.

Because LAIV is delivered mucosally, the induction of a local immune response may be relevant in its protective efficacy; we therefore measured antigen‐specific IgA responses in nasal samples against all strains by ELISA and normalized these to total IgA in the samples. Variable sample recovery and low amounts of total IgA meant that responses could not be assessed against all antigens for all participants (Table 2). There was a significant increase in nasal IgA titre to the H3N2, B/Bris and B/Phu components (Fig. 1a), but no change in the response to H1N1. The geometric mean fold‐change in the nasal H3N2 IgA response [2·3 (1·7–3·1)] was significantly greater than the fold‐change in the nasal IgA to H1N1 [1·0 (0·8–1·3), *P* < 0·001] or B/Phu [1·4 (1·1–263), *P* = 0·0048] (Fig. 1b). We also measured the influenza‐specific IgA in the serum of the 39 children who gave blood samples. No differences were seen in antigen‐specific serum IgA (Fig. 1c) or fold‐change between pre‐ and post‐vaccination serum samples (Fig. 1d). Nasal IgA seroconversion rates of the four vaccine strains, defined as a fourfold increase in absolute nasal IgA antibodies pre‐ and post‐vaccination, were as follows; 7·4% (7/95) for H1N1, 29·5% (28/95) for H3N2, 28·2% (24/85) for B/Bris and 11·7% (9/77) for B/Phu. No recipient had a fourfold increase in serum IgA to all four strains.

**Table 2:** Nasal swab IgA responses to vaccination

Antigen	Samples analysed	Pre‐immunization	Post‐immunization	Fold‐change	Proportion with fourfold increase
H1N1 (A/California/7/2009 pdm‐like)	95/103 (92%)	0·042 (0·033–0·053)	0·041 (0·033–0·053)	1·0 (0·80–1·3)	7/95
H3N2 (A/Switzerland/9715293/2013)	95/103 (92%)	0·024 (0·019–0·030)	0·057 (0·043–0·075)	2·3 (1·7–3·1)	28/95
B: Yamagata‐like (B/Brisbane/60/2008)	85/103 (83%)	0·025 (0·020–0·032)	0·047 (0·035–0·063)	1·9 (1·4–2·5)	24/85
B: Victoria‐like (B/Phuket/3073/2013)	77/103 (75%)	0·015 (0·013–0·017)	0·020 (0·016–0·024)	1·4 (1·1–1·6)	9/77

Data presented as geometric mean of antigen‐specific titre as a percentage of total immunoglobulin (IgA) and 95% confidence intervals.

### Factors affecting nasal IgA responses

**Figure 2:**
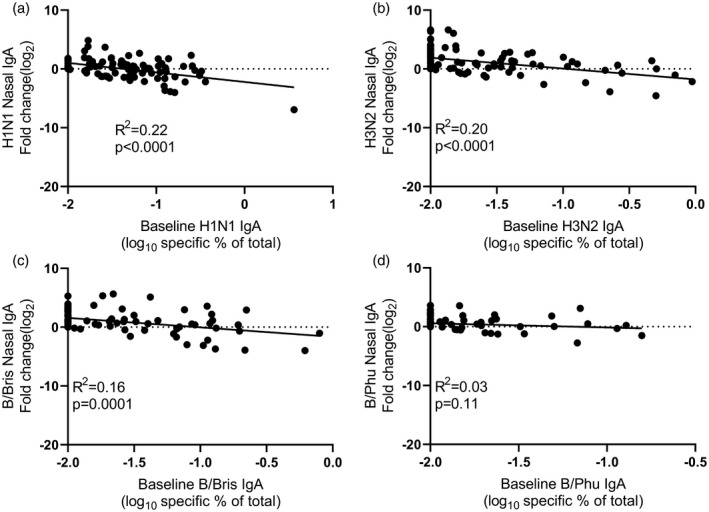
Relationship between baseline nasal immunoglobulin (Ig)A titre and fold change in nasal IgA response. Nasal IgA titre at baseline was compared to the fold change in response to H1N1 (a), H3N2 (b), B/Bris (c) and B/Phu (d) antigens.

**Figure 3:**
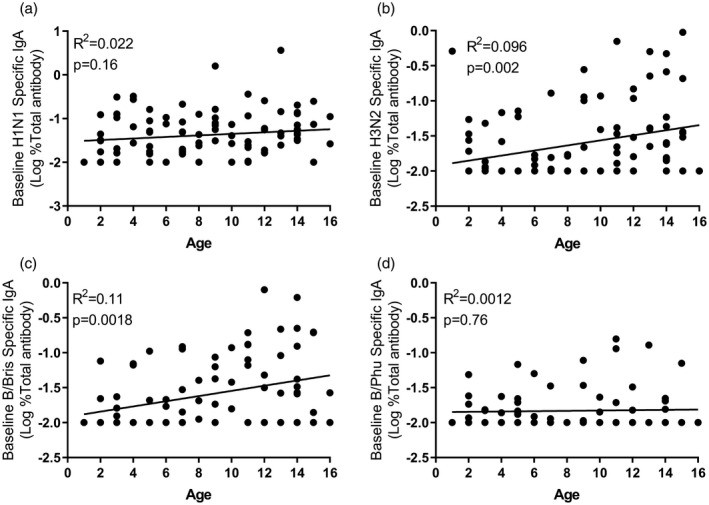
Relationship between age and baseline nasal immunoglobulin (Ig)A. Age was compared to the nasal IgA titre at baseline to H1N1 (a), H3N2 (b), B/Bris (c) and B/Phu (d) antigens for all children.

Given the significant differences in nasal IgA responses to the different antigens, we performed a *post‐hoc* analysis to evaluate the factors that might cause these differences. One possibility is that pre‐existing immunity may reduce vaccine response. We investigated whether there was a link between baseline nasal IgA and fold change in nasal IgA after immunization. There was a weak but significant negative relationship between baseline nasal IgA and fold‐change in response for H1N1, H3N2 and B/Bris (Fig. 2). As baseline nasal IgA response may reflect the history of virus exposure which is dependent on age, we compared the age of the child at immunization with the baseline nasal IgA (Fig. 3). We found a significant, but again very weak, correlation between age and baseline nasal IgA for H3N2 (Fig. 3b) and B/Bris (Fig. 3c), but no correlation for nasal IgA responses to H1N1 (Fig. 3a) or B/Phu (Fig. 3d).

**Figure 4:**
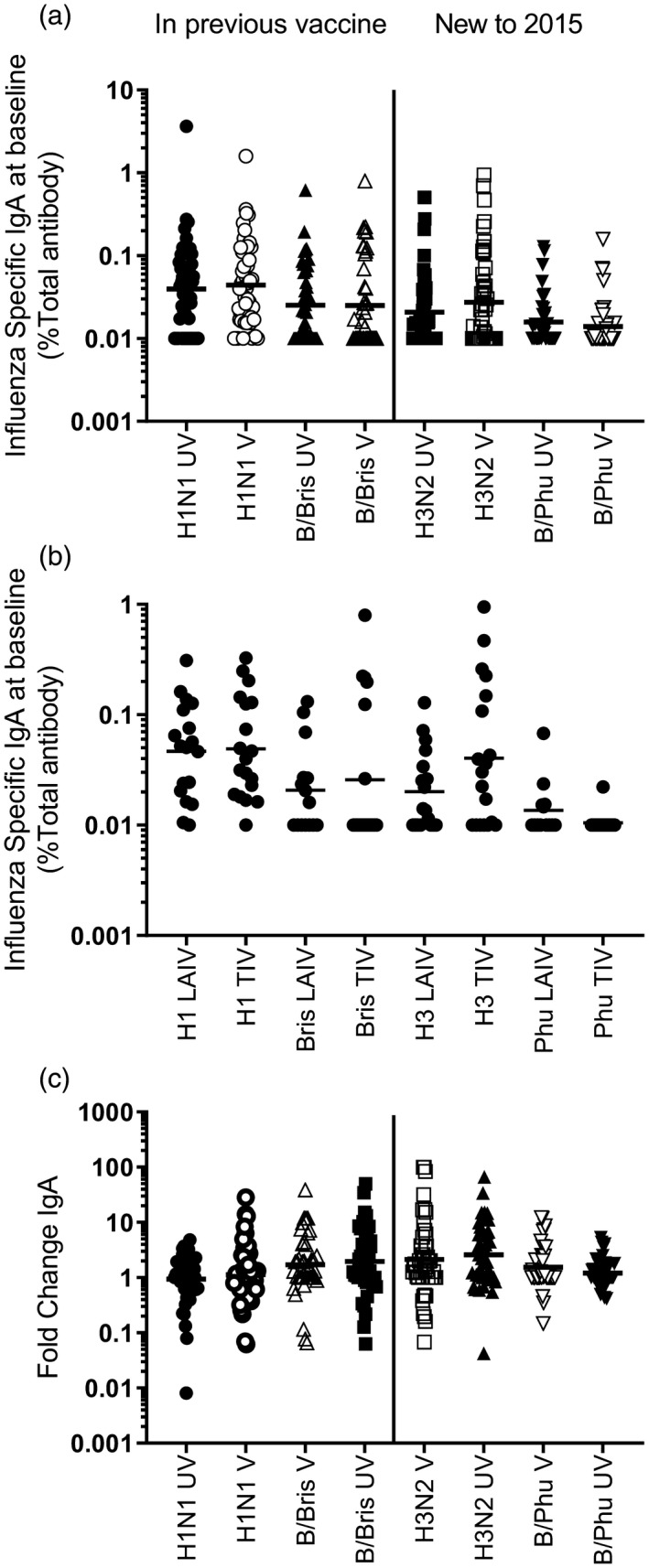
Impact of influenza vaccination history on nasal immunoglobulin (Ig)A. Children grouped by previous vaccination status, vaccinated (closed symbols) or no previous vaccine (open symbols). Baseline nasal IgA prior to immunization comparing previously immunized and immunized (a) comparing previously immunized by vaccine type before – live attenuated influenza vaccine (LAIV) or IIV (b). Nasal IgA fold change (c) points represent individuals, lines geomean.

We also investigated whether previous influenza vaccination affected nasal IgA. The children were grouped by whether or not they previously had received influenza vaccination. Two of the strains in this study have been previously included in LAIV: A/California/7/2009 pdm‐like and B/Brisbane/60/2008, and two of the strains were newly included in the 2015–16 vaccine formulation: A/Switzerland/9715293/2013 and B/Phuket/3073/2013. There was no difference in the baseline nasal IgA titres to any of the vaccine strains when comparing vaccine‐naive children and previously vaccinated children (Fig. 4a). We did not identify a difference in baseline nasal IgA titres in children who had previously received LAIV compared to IIV (Fig. 4b). There was no significant difference in fold‐change of nasal IgA when children were grouped by previous vaccination history (Fig. 4c).

**Figure 5:**
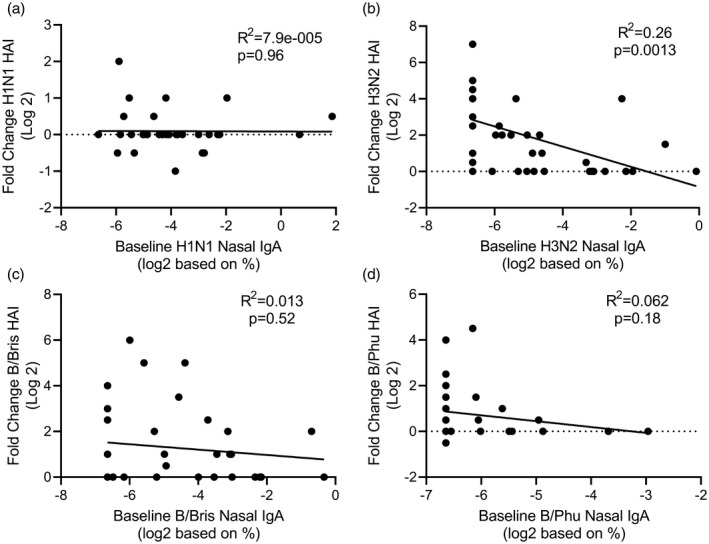
Relationship between baseline nasal immunoglobulin (Ig)A and fold change in haemagglutination inhibition assay (HAI) response. Nasal IgA titre at baseline was compared to the fold change in HAI response to H1N1 (a), H3N2 (b), B/Bris (c) and B/Phu (d) antigens.

We have previously observed that baseline nasal IgA affected the number of days of viral shedding during human influenza challenge infection 15. As a link between LAIV viral shedding and immunogenicity has been proposed 16, we investigated whether baseline nasal IgA affected vaccine immunogenicity. We looked at fold‐change in HAI as a surrogate measure of immunogenicity in a subset of children. There was no relationship between baseline nasal IgA and HAI fold‐change for H1N1 (Fig. 5a), B/Bris (Fig. 5b) or B/Phu (Fig. 5c). However, children with a higher level of H3N2‐specific nasal IgA at baseline had a significantly lower HAI fold‐change (Fig. 5d).

## Discussion

In this study, we measured local, nasal IgA responses following vaccination of children with quadrivalent LAIV in the 2015–16 season. There was an increase in the nasal IgA to both B strains and H3N2, but not H1N1. The fold change in response to nasal IgA was inversely proportional to baseline nasal IgA titre and there was a correlation between age and baseline titre to the H3N2 and B/Bris strains. Based on these data, we conclude that LAIV is able to induce a nasal IgA response, but the response varies among the different strains incorporated in the vaccine: this is similar to the pattern seen in HAI responses 17.

The pH1N1 strain (A/California/7/2009 pdm‐like: A/Bolivia/559/2013) did not induce a significant change in nasal IgA. This matches observations of LAIV vaccination in the same time‐period where changes in (serum) HAI responses to B antigens were greater than to H1N1 17. These differences in immune response may reflect reduced vaccine efficacy for the H1N1 component of LAIV 18. It is not clear why individual strains behave differently. It has been suggested that the incorporation of the pH1N1‐derived haemagglutinin has an effect on vaccine thermostability 19. This might be exacerbated by the multivalent nature of LAIV: with the current recommendation to include four strains (H1N1, H3N2 and two influenza B strains), fitter strains may outcompete or induce an anti‐viral state that inhibits the take of the other strains. Such an effect is seen with trivalent oral polio vaccine 20, 21 in which the type 2 strain is the most immunogenic and interferes with the take of types 1 and 3 in the vaccine. A recent study has shown that changing the H1N1 strain in the St Petersburg backbone vaccine has led to increased vaccine shedding and immunogenicity 16. Further studies are required to investigate the link between shedding and immunogenicity.

The other aspect we investigated in the current study was the impact of pre‐existing immunity on vaccine immunogenicity. It has been suggested that repeated vaccination in the United States may have been a contributing factor to reduced LAIV vaccine efficacy. Given the UK programme, it is important to understand whether repeated vaccination may contribute to reduced vaccine efficacy. We found that higher baseline nasal IgA titres were associated with significantly lower fold‐changes in nasal IgA for some vaccine subtypes. For H3N2 and B/Bris, there was a correlation with age and baseline titre; this was not seen for H1N1 and B/Phu. Differences in baseline nasal IgA cannot be explained by previous vaccine exposure alone, as there was no difference in baseline titre when children were compared by vaccination history. This suggests that other factors are affecting the differences in response to the different strains in the vaccine.

Recovery was limited for some of the mucosal samples, and we were unable to analyse all four strains for all the volunteers. We have previously looked at other methodologies for sampling the nasal mucosa for antibody and observed that synthetic absorptive matrix (SAM) strips gave the greatest concentration yield of antibody 22. The current study suggests that, for larger community‐based studies, posting samples on flocked swabs is effective. The nasal swabbing was considerably less invasive than collecting blood for HAI; hence, we received a greater number of samples for nasal IgA analysis. Replacing blood collection with other, less invasive sampling methods that can be performed remotely by the study recipient or carer may lead to greater response rates, and is likely to explain the higher rate of families willing to participate in the nasal sampling than the serological sampling in our study, although compliance with the follow‐up nasal sample self‐taken at home was disappointing. We did not find any obvious differences in those children with paired samples compared to the overall cohort, so this was not expected to bias the study.

It remains unclear as to the best correlate of protection for LAIV. Other studies have linked protective efficacy of LAIV to nasal IgA in children 23 and suggested that nasal IgA could be considered as a co‐correlate of protection. That we saw no increase in nasal IgA fold to the H1N1 strain after vaccination supports the relatively lower efficacy to H1N1 of LAIV observed elsewhere. We have recently observed that mucosal IgA correlates with reduced viral shedding after human influenza challenge 15. This may be important when considering the herd immunity value of LAIV: if vaccine‐induced nasal IgA reduces the time vaccinated individuals shed virus if they become infected, this may prevent spread to older, more at‐risk populations, as seen with pneumococcal conjugate vaccine 24. While we were unable to directly assess vaccine efficacy, comparing our data to surveillance data for the same time‐period of the study is of interest. The 2015–16 influenza season, when our study was performed, was characterized by influenza A(H1N1)pdm09 viruses followed by B/Brisbane/60/2008 (the influenza B/Victoria lineage virus) later in the season 25. Vaccine efficacy (VE) for LAIV in 2015–16 reflected what we saw with the antibody response – H1N1 did not induce a significant increase in nasal IgA and the VE was only 41·5%, B/Bris induced a significant increase in nasal IgA and the VE was 81·4%, compared to 56·4% for IIV. However, this inference is limited due to a lack of VE data with respect to H3N2 or B/Phu in the same time‐period. Because the enrolment occurred throughout the influenza season, some of the changes in titre seen may be caused by infection rather than immunization, but the majority of immunizations were given early in the season, reducing the likelihood of this.

In the current study, we investigated the IgA response in serum and nasal secretions to quadrivalent LAIV in children. We observed differences in responses between the four strains included in the vaccine in 2015–16. As seen previously with HAI responses in serum 17 the H1N1 strain was less immunogenic than other strains. We still do not fully understand how LAIV protects against influenza infection, and what role nasal IgA plays within this. Understanding the mechanisms of LAIV‐induced protection is important for future vaccine development and implementation.

## Disclosures

There were no competing interests.

## Author contributions

P. J. T. was Chief Investigator of the study. A. F. A. collected samples. M. E. C., R. R. J., V. G. and K. H. performed laboratory analysis. M. E. O'D. and N J. A. performed statistical analysis. M. Z., E. M., P. J. T. and J. T. designed the study and wrote the manuscript.

## Supporting information


**Fig. S1.** Recovery of influenza specific nasal IgA is not affected by posting samples. Adult volunteers, had nasal samples collected by flocked swab. Samples were either processed directly from the nose or posted via the internal mail system. Having processed the samples they were analysed for H1N1 (a) or H3N2 (b) specific nasal IgA by ELISA. *n* = 6.
