# Supplementary figures and images for: Differences in nasal immunoglobulin A responses to influenza vaccine strains after live attenuated influenza vaccine (LAIV) immunization in children
Source: Clin Exp Immunol. 2019 Nov 15;199(2):109–18. doi: 10.1111/cei.13395 (PMC6954673; doi:10.1111/cei.13395)

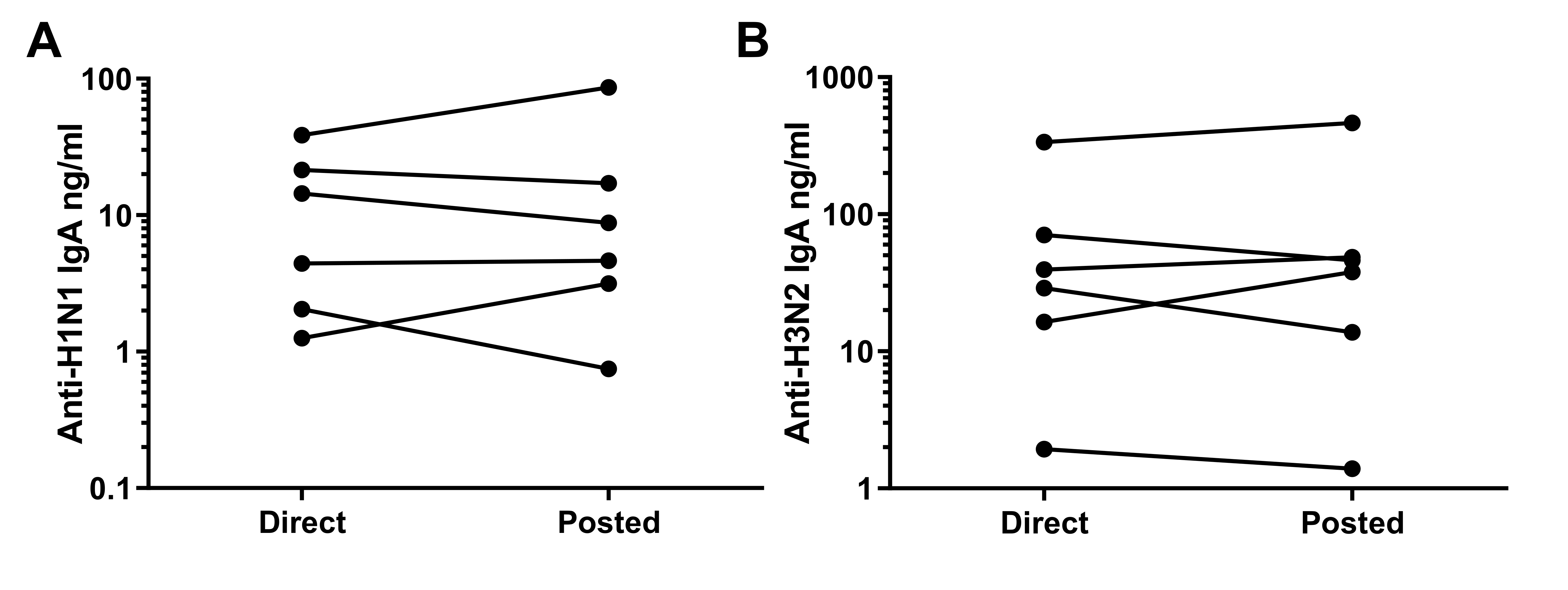

Supplement: Supplementary file 1 — Fig. S1. Recovery of influenza specific nasal IgA is not affected by posting samples. Adult volunteers, had nasal samples collected by flocked swab. Samples were either processed directly from the nose or posted via the internal mail system. Having processed the samples they were analysed for H1N1 (a) or H3N2 (b) specific nasal IgA by ELISA. n = 6. [file CEI-199-109-s001.tif]
